# Implant survivorship, functional outcomes and complications with the use of rotating hinge knee implants: a systematic review

**DOI:** 10.1186/s43019-022-00138-2

**Published:** 2022-03-04

**Authors:** Joshua Xu, Lennart von Fritsch, Shiraz A. Sabah, Andrew J. Price, Abtin Alvand

**Affiliations:** 1 Nuffield Orthopaedic Centre, Windmill Rd, Oxford, OX3 7LD UK; 2 Nuffield Department of Orthopaedics, Rheumatology and Musculoskeletal Sciences, Botnar Research Centre, University of Oxford, Old Road, Oxford, OX3 7LD UK

**Keywords:** Rotating hinge knee, Total knee replacement, Reoperation, Revision, Patient-reported outcome measures

## Abstract

**Background:**

With more complex primary and revision total knee arthroplasty procedures there is often the need to use more constrained prostheses. This study aims to investigate patient-relevant outcomes following primary and revision rotating-hinged total knee arthroplasty.

**Methods:**

Electronic searches were performed using four databases from their date of inception to January 2021. Relevant studies were identified, with data extracted and analysed using PRIMSA guidelines.

**Results:**

Nineteen studies were included, producing a cohort of 568 primary and 413 revision rotating hinge total knee arthroplasties (TKAs). Survival was assessed at 1-, 5-, and 10-year post-implantation. Sensitivity analyses based on person-time incidence ratios (PTIRs) were prespecified for studies not reporting survival at these timepoints. From the primary hinge TKA cohort, the median survival at 1 year was 93.4% and at 10 years it was 87%. The PTIR at long-term follow-up of this primary cohort was 1.07 (95% CI 0.4–1.7) per 100 person-years. From the revision hinge TKA cohort, the median survival at 1 year was 79.6%, and at 10 years it was 65.1%. The PTIR at long term-follow-up of this revision cohort was 1.55 (95% CI 0.9–2.3) per 100 person-years. Post-operative flexion range of motion (ROM) was 110° for primary hinge TKA and 103° for revision hinge TKA. Compared with baseline, the Knee Society Score (KSS) and Knee Society Function Score (KSFS) improved for both groups post-operatively (primary: KSS 17 to 86, KSFS 28 to 58; revision: KSS 37 to 82, KSFS 34 to 61).

**Conclusion:**

The quality of the evidence for patient-relevant outcomes following hinged knee arthroplasty was limited. While there is the potential for high early revision rates, where successful, large functional benefits may be achieved.

## Introduction

Total knee arthroplasty (TKA) is a highly effective operation for the management of knee osteoarthritis [1]. The success of this surgery has resulted in a rise in demand from patients [2], including those who are younger and more active, and those with deformity who would not have previously been considered candidates for surgery. The requirement for complex primary, revision (and re-revision) TKA has risen accordingly [3]. For these patients, a more constrained knee replacement may be needed to provide optimal reconstruction.

When selecting an implant for reconstruction, one important principle is to select the least-constrained device that is considered appropriate [4]. The rationale is to minimise stresses at the bone–cement–implant interface and subsequent failure due to aseptic loosening [5]. Less constrained devices typically require a more conservative bone resection than hinged implants, and place lower demands on a stem for fixation, providing greater options for future reconstruction should it be necessary. However, in some cases, this must be balanced against the risk of instability, which may require subsequent revision surgery or provide a source of pain, poor function and patient dissatisfaction [6, 7].

For primary knee replacement, hinge-type devices are rarely needed, accounting for around 0.2% of procedures [3]. A recent study identified limited, specific indications for primary hinged knee replacement, and recommended that they are reserved mainly for elderly patients [8]. These indications included insufficiency of collateral ligaments, valgus or varus deformity, neuropathic arthropathy and significant bony defects [8]. For revision knee replacement, hinge-type devices are required in a greater proportion of cases due to the greater prevalence of ligamentous incompetence and bone loss [7].

Most studies reporting on the outcomes of hinge-type knee replacements have been small, retrospective observational studies focusing on implant survivorship. Few studies have provided information on other patient-relevant outcomes, such as pain, joint function and health-related quality of life [9]. In addition, much of the literature refers to early, highly constrained, fixed hinge designs which may not be relevant to current practice. More modern, rotating hinge implants combine flexion–extension with rotation of the femur on the tibial component. This allows more physiological movement on the prosthetic knee joint, reducing the stress placed on the implant, when compared with fixed hinge designs [6, 10, 11].

The aim of this study was to systematically review the evidence for patient-relevant outcomes following modern, rotating-hinge TKA. We investigate implant survivorship, joint function, health-related quality of life and complications following surgery. We report findings separately for primary and revision TKA, and summarise information reported by international and regional joint registries.

## Methods

### Search strategy

The Preferred Reporting Items for Systematic Reviews and Meta-Analyses (PRISMA) guidelines were followed for this study. Electronic database searches were performed using PubMed, Ovid Medline, Cumulative Index of Nursing and Allied Health Literature (CINAHL) and Cochrane CENTRAL from their dates of inception to January 2021. The search strategy is provided in Appendix 1. The sensitivity of the search strategy to detect studies on hinged implants was maximised by including the names of common brands as search terms. The reference list of all retrieved articles was manually reviewed to further identify potentially relevant studies. National and regional joint registries listed in The International Society of Arthroplasty Registries (ISAR) were reviewed for data on rotating hinges.

### Selection criteria

Eligible studies for this systematic review included patients undergoing primary or revision TKA using a rotating hinge implant. Included studies were required to report post-operative knee function. If multiple studies reported outcomes from the same cohort, data from the longest follow-up period was included for quantitative analysis. If studies reported survivorship at multiple follow-up periods, these were all included in our survivorship analysis. Neoplastic indications for rotating hinge TKA were excluded. All publications included were limited to those in the English language and involving human subjects. Conference presentations, case reports, reviews, editorials, and expert opinions were excluded. Studies with mixed primary and revision cohorts were excluded.

### Data extraction

Two investigators (J.X and L.F) independently reviewed and extracted data from the retrieved articles. Discrepancies between the two reviewers were resolved by discussion with senior authors. Data were extracted on study year, country, number of patients undergoing primary and revision TKA, and indication for surgery.

The primary outcome measures were implant survivorship at 1-, 5- and 10-years following rotating hinge knee arthroplasty. Construct survival estimates and associated confidence intervals were extracted for these time points to allow pooling with meta-analysis if appropriate. Person-time incidence ratios (PTIR) were used to assess the incidence of implant failure in studies not reporting survival at these time points. Person-time (PT) was calculated by multiplying the number of cases and the mean follow-up. PTIRs per 100 person-years were then calculated based on the number of construct failures over the follow-up period. PTIRs were grouped by mean follow-up duration into short-term (< 1 year), medium term (1–5 years) and longer term (> 5 years).

Secondary outcome measures were knee range of motion, knee function and surgical complications. Knee range of motion was measured in degrees as the arc of movement between maximum knee flexion and extension. Knee function included both surgeon-completed [e.g. Knee Society Score (KSS), Knee Society Function Score (KSFS)] and patient-completed scores [e.g. Oxford Knee Score (OKS)]. The KSS and KSFS were scored from 0 (worst) to 100 (best), and OKS from 0 (worst) to 48 (best). The number of surgical complications (including reoperations not classified as revisions or re-revisions) was recorded according to each of the time periods specified.

### Data synthesis

Our statistical analysis plan prespecified a decision to be taken on whether or not to perform meta-analysis based on the body of evidence available after data extraction. Due to the clinical diversity of the included studies, incomplete reporting of effect estimates and uncertainty, and the methodological and statistical heterogeneity observed, we decided to perform systematic review without meta-analysis (SWiM) [12]. This approach is useful to report the range and distribution of effects when the average effect size cannot be calculated through meta-analysis. We selected to present medians and ranges for each of the available outcome measures. An important limitation of these estimates is that they do not account for study size.

### Study quality

The quality of included studies was assessed using a non-summative four-point system developed by Wylde et al. [13] to rate studies on joint replacement. Studies were rated based on the inclusion of consecutive cases, representativeness (whether the study was multi-centre), adequacy of follow-up (defined as < 20% loss to follow-up) and minimisation of confounding (defined as use of multivariate analysis).

## Results

### Search results

**Fig. 1:**
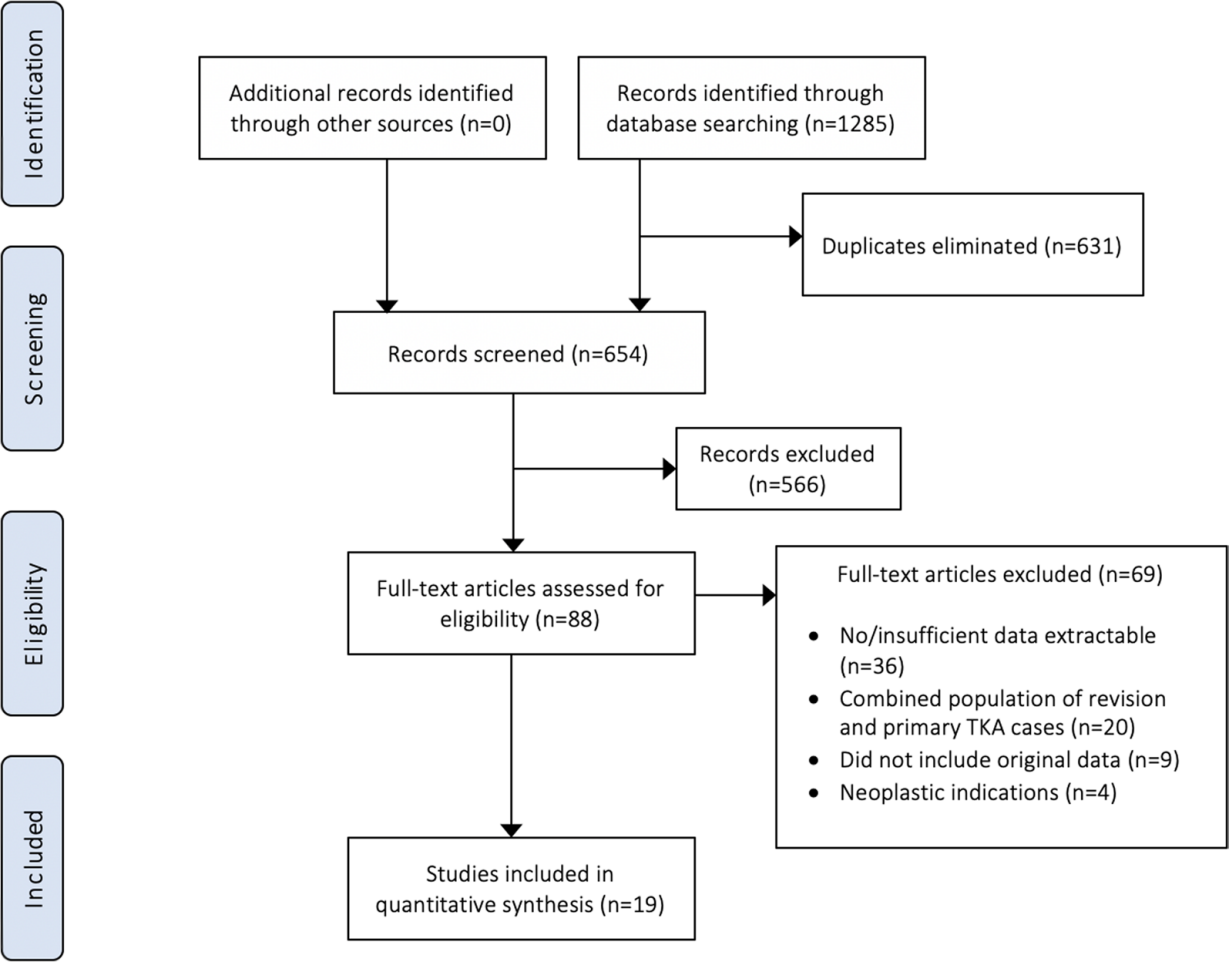
PRISMA flow chart of systematic review on clinical outcomes and complications of rotating hinge TKA

An initial search led to the identification of 1285 references (Fig. 1). After duplicate studies were removed, a total of 654 studies remained for screening. A further 566 studies were excluded following abstract screening, leaving 88 studies for full-text analysis. A total of 19 studies [14–32] were eligible following application of the inclusion criteria. Manual searching of references in each of the full-text articles did not yield further studies for inclusion.

### Characteristics of the included studies

No randomised controlled trials were identified. Nineteen observational studies (18 retrospective, 1 prospective) were included. A total of 915 patients with 981 total knee operations were extracted from the 19 studies. There were 568 hinge TKAs performed for primary TKA, and 413 performed for revision TKA. Seven studies reported on primary TKA and 12 studies on revision TKA. The study year ranged from 2000 to 2019, and patients had a median follow-up of 79.5 months (range 28–180 months). The median patient age was 69.7 years (range 65–79 years). The median proportion of females was 67.1% (range 39.3–100%). The study characteristics are summarised in Table 1.

**Table 1:** Characteristics and demographics of included studies

Author	Year	Study period	Country	Study type	Implant type	No. knees	No. patients	Females %	Mean age (range)	FU – mean years (range)
Revision cases										
Rodriguez	2015	–	Spain	OS, R	Endo-Model	96	96	75.0	79.0 (75–86)	7.3 (5–10)
Neumann	2011	2005–2007	Austria	OS, R	NexGen	24	24	41.7	67.0 (40–87)	4.7 (3–5)
Gudnason	2011	1991–2003	Sweden	OS, R	Endo-Model	42	38	68.4	72.0 (55–88)	8.8 (8–18)
Pradham	2004	1996–2001	UK	OS, R	Endo-Model	51	50	58.0	70.3 (39–85)	4.0 (2–6)
Bistolfi	2013	1991–2004	Italy	OS, R	Endo-Model	53	50	66.0	69.7 (45–85)	12.9 (7–20)
Barrack	2000	–	USA	OS, R	S-ROM Noiles	14	13	46.2	69.0 (34–80)	4.3 (2–6)
Abdelaziz	2019	2007–2009	Germany	OS, R	Endo-Model	25	25	48.0	65.0 (40–70)	10.5 (10–12)
Back	2008	–	UK	OS, R	SMILES	32	30	63.3	67.0 (46–86)	4.8 (5–11)
Baier	2013	2003–2007	Germany	OS, R	TC3/S-ROM Noiles	78	78	66.7	69.0 (53–84)	6.8 (5–9)
Bistolfi	2012	2002–2008	Italy	OS, R	NexGen RH	31	29	82.8	72.8 (43–81)	5.0 (3–8)
Joshi	2008	1993–2002	Spain	OS, R	Endo-Model	78	78	80.8	72.0 (53–88)	7.8 (5–11)
Pour	2007	1997–2003	USA	OS, R	Kinematic and Finn	44	43	67.4	71.8 (55–88)	4.2 (2–8)
Primary cases										
Yang	2011	1992–2000	Korea	OS, R	Endo-Model	50	40	100	72.0 (59–82)	15.0 (10–18)
Rahman	2015	1996–2013	UK	OS, R	SMILES	14	13	76.9	66.0 (51–84)	6.0 (1–13)
Lozano	2012	2006–2009	Spain	OS, R	Endo-Model	111	104	80.8	72.8	2.3
Kowalczewski	2014	2001–2003	Poland	OS, P	Modular	12	12	58.3	67.5 (43–83)	(10–12)
Petrou	2006	1987–1995	Greece	OS, R	Endo-Model	100	80	-	70.0 (56–85)	11.0 (7–15)
Bistolfi 2	2013	1992–1995	Italy and France	OS, R	Endo-Model	98	84	83.3	69.1 (34–84)	14.5 (13–16)
Leng	2018	2006–2012	China	OS, R	Endo-Model	28	28	39.3	72.5 (60–81)	6.5 (4–10)

*n* number, *%* percentage, *OS* observational, *R* retrospective, *P* prospective, – not reported, *FU* follow-up

### Indications for surgery

For the seven studies reporting on primary TKA, osteoarthritis was the most common indication for surgery, accounting for a median of 68.7% of cases (range 25–90.1%). Rheumatoid arthritis was the only other surgical indication specified.

For the 12 studies reporting on revision TKA, the most common indication for surgery was aseptic loosening, with a median of 57.1% (range 40.0–100%). The median reported rates for other indications were infection 21.8% (range 12.9–45.1%), instability 21.4% (range 6.3–100%), implant wear/breakage 9.2% (range 3.2–26.0%), bone loss 37.8% (range 14.3–61.4%) and periprosthetic fracture 2.0% (range 1.3–90.1%).

### Implant survival

**Fig. 2:**
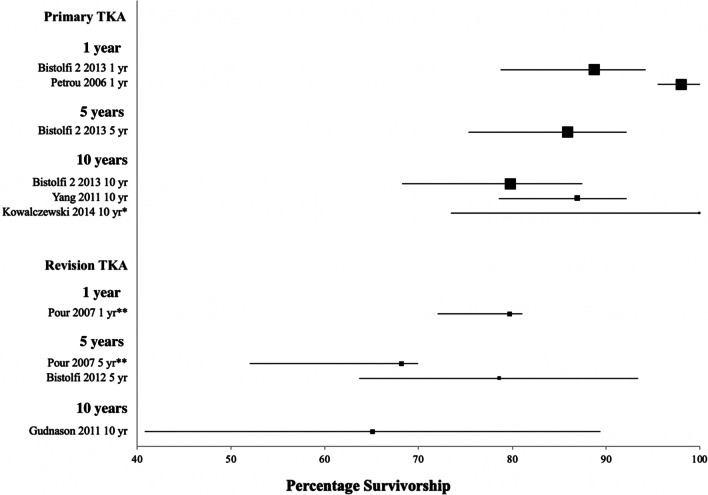
Forest plot for survivorship of hinge knee prosthesis at 1-, 5- and 10-years post-operation. All values are presented with 95% confidence intervals and squares weighted to population size. *95% CI calculated using CII proportions in Stata. **95% CI calculated from Kaplan–Meier curve using Web Plot Digitizer

There was heterogeneity in the reporting of survivorship for hinged TKAs. For the seven studies reporting on primary TKA, four studies (57.1%) reported on implant survivorship at the prespecified fixed timepoints, and five studies (71.4%) provided data from which PTIRs could be calculated. The median survival at 1 year was 93.4% (range 88.7–98%) (two studies [28, 29]), at 5 years was 85.9% (one study [29]) and at 10 years was 87% (range 79.8–100%) (three studies [25, 27, 29]). This is illustrated in the forest plot (Fig. 2). The longest follow-up was at 15 years, reported as 75.8% by Bistolfi et al [29]. For PTIR, all studies fell into the long-term follow-up group, where mean PTIR was 1.07 [95% confidence interval (CI) 0.4–1.7] per 100 person-years.

For the 12 studies reporting on revision TKA, 3 studies (25.0%) reported implant survivorship at the prespecified fixed timepoints and 9 studies (75%) provided data from which to calculate PTIRs. The median survival at 1 year was 79.6% (one study [23]), at 5 years was 77.0% (range 68.2–85.7%) (two studies [21, 23]) and 10 years was 65.1% (one study [16]). This is also illustrated in the forest plot (Fig. 2). The longest follow-up was at 12.5 years, reported as 80.4% by Bistolfi et al [18]. Overall PTIR was 1.74 (95% CI 1.1–2.4) per 100 person-years. PTIR at medium follow-up was 2.12 (95% CI 0.7–3.5) per 100 person-years and at long-term it was 1.55 (95% CI 0.9–2.3) per 100 person-years.

### Functional outcomes

For the seven studies reporting on primary TKA, the instruments used to measure knee function were flexion ROM in five studies, KSS in three studies, KSFS in two studies and OKS in one study (Table 2). For post-operative flexion range of motion, the median was 110° (range 102–120°). The median KSS pre-operatively was 7.4 (range 11.4–38.0) and improved post-operatively to 86.2 (range 73.0–93.4). The KSFS pre-operatively was 27.9 (range 19.7–36.0), and improved post-operatively to 58.4 (range 47.0–69.7). The OKS preoperatively was 11.6 and improved post-operatively to 31.5 [24].

**Table 2:** Functional and patient-reported outcomes measures

Author	Year	Post-operative flexion ROM – mean (range)	KSS – mean (range)		KSFS – mean (range)		OKS – mean (range)	
			Pre-op	Post-op	Pre-op	Post-op	Pre-op	Post-op
Revision cases								
Rodriguez	2015	120	37	79	34	53	–	–
Neumann	2011	116 (90–125)	25	91 (82–97)	35 (15–45)	85 (60–100)	–	–
Gudnason	2011	108 (100–120)	–	85 (73–96)	–	29 (0–100)	–	–
Pradham	2004	–	–	–	–	–	–	–
Bistolfi	2013	103 (97–108)	–	–	–	–	–	–
Barrack	2000	93 (70–125)	41 (6–81)	131 (104–160) -		–	–	–
Abdelaziz	2019	92 (30–120)	–	–	–	–	–	-
Back	2008	88 (5–110)	26 (15–48)	68 (40–85)	27 (10–55)	–	–	-
Baier	2013	–	57 (28–80)	71 (42–97)		–	–	–
Bistolfi	2012	114 (108–121)	–	–	–	–	–	–
Joshi	2008	102 (50–130)	38 (10–75)	86 (44–98)	33 (0–85)	61 (20–100)	–	–
Pour	2007	–	29 (0–89)	74 (33–86)	40 (5–91)	43 (30–100)	–	–
Primary cases								
Yang	2011	102	38	73	36	47	–	–
Rahman	2015	–	–	–	–	–	12 (4–18)	32 (18–39)
Lozano	2012	120 (100–120)	–	–	–	–	–	–
Kowalczewski	2014	110 (80–120)	17	86	–	–	–	–
Petrou	2006	120 (100–130)	11 (0–46)	93 (75–100)	20 (0–50)	70 (15–100)	–	–
Bistolfi 2	2013	110	–	–	–	–	–	–
Leng	2018	–	–	–	–	–	–	–

*ROM* range of motion, *KSS* Knee Society Score, *KSFS* Knee Society Function Score, *OKS* Oxford Knee Score, – not reported

For the 12 studies reporting on revision TKA, the instruments used to measure knee function were flexion ROM in 9 studies, KSS in 8 studies and KSFS in 8 studies. The OKS was not reported. For post-operative flexion range of motion, the median was 102.6° (range: 88–120°). The KSS pre-operatively was median 37.0 (range 25.0–56.9) and improved post-operatively to 82.0 (range 68.0–131.0). The KSFS preoperatively was 34.0 (range 27.0–40.0), and improved post-operatively to 61.1 (range 29.0–85.0).

### Complications

Eighteen studies (94.7%) reported on post-operative complications, with only two studies reporting some complications with time-to-event data [23, 24]. The remainder of the studies presented simple counts of complications over the study period, thus meaningful narrative or quantitative summary of complication rates could not be made. Appendix 2 shows the summary of complications data for each study.

### Quality of the included studies

From the included studies, 11 (57.9%) reported that they included consecutive patients, 0 (0.0%) studies were reported to be multi-centre, 15 (78.9%) studies reported adequate follow-up (> 80% of original cohort) and 0 (0.0%) studies minimised confounding using multivariate analysis. These results are summarized in Table 3.

**Table 3:** Assessment of methodological quality of included studies

Author	Year	Inclusion of consecutive patients	Representativeness (multi-center)	Adequate follow-up of > 80% (follow-up%)	Minimisation of confounding (multivariate analysis)
Revision cases					
Rodriguez	2015	–	No	Yes	No
Neumann	2011	Yes	No	Yes	No
Gudnason	2011	Yes	No	No	No
Pradham	2004	–	No	Yes	No
Bistolfi	2013	–	No	No	No
Barrack	2000	Yes	No	Yes	No
Abdelaziz	2019	–	No	Yes	No
Back	2008	Yes	No	Yes	No
Baier	2013	Yes	No	Yes	No
Bistolfi	2012	Yes	No	–	No
Joshi	2008	Yes	No	Yes	No
Pour	2007	–	No	Yes	No
Primary cases					
Yang	2011	No	No	Yes	No
Rahman	2015	Yes	No	Yes	No
Lozano	2012	Yes	No	Yes	No
Kowalczewski	2014	Yes	No	Yes	No
Petrou	2006	No	No	Yes	No
Bistolfi 2	2013	–	No	No	No
Leng	2018	Yes	No	Yes	No

– not reported

### Registry studies

The International Society of Arthroplasty Registries (ISAR) includes 36 registries making up 24 national, 6 regional and 6 other registries. Only three registries [German registry (EPRD) [33], Finnish registry (FAR) [34] and National Joint Registry (NJR) [35]] publicly reported implant survivorship for modern, rotating hinge knee implants. Seventeen (47.2%) registries did not provide a publicly available report or not in the English language. Sixteen (44.4%) registries did not specify survivorship for modern rotating-hinge implants. This included seven registry reports which were excluded because they provided pooled outcomes for modern rotating-hinges and older fixed-hinge designs together. For specific brands of rotating hinge knee replacements, revision probabilities are provided in Appendix 3. The latest NJR report [3] stated that 2 out of 11 outlier implants for primary knee replacement reported to the Medicines and Healthcare Products Regulatory Agency (MHRA) were rotating hinge knee replacements.

## Discussion

This study has critically summarised patient-relevant outcomes following modern rotating hinge primary and revision knee arthroplasty. The evidence base consisted of low quality, small, single-centre, case series, with 568 primary hinge TKA procedures and 413 revision hinge TKA procedures contributing to this review. The revision rate for primary hinge TKA from the included studies ranged from 2% to 11% at 1 year and 0% to 20% at 10 years. For revision hinge TKA, the rates of re-revision at 1 year was only reported in one study to be 20%, and ranged from 12% to 35% at 10 years. Three joint replacement registries (the German registry [33], Finnish registry [34] and National Joint Registry [35]) reported 1-year implant survivorship for modern rotating hinge implants after complex primary TKA. The reported revision rates at 1-year ranged from 1.4% to 4.8%. Only the FAR and NJR reported 10-year implant survivorship, with revision rates ranging from 8.3% to 14.0%. Re-revision data was not available for use of modern rotating hinge knee implants in revision TKA.

The evaluation of joint range of movement or function was required for inclusion in this review. Only one study [24] used a patient-completed score (the Oxford Knee Score) to assess joint function, with the remaining studies using clinician-completed instruments (the KSS and KSFS) or range of motion only. There was a large improvement in joint function from pre-operative baseline to post-operative follow-up for both primary and revision hinged knee replacements. Data on medical and surgical complications were poorly reported. The majority of studies simply reported counts of complications over their respective study periods. This is an inappropriate method for calculating complication rates which need to be paired with time data (e.g. a fixed time point, such as 90-days post-operation) [36].

The main strength of this study is its systematic evaluation of the current literature on hinged knee replacements for primary and revision surgery. Due to the clinical diversity of patients and poor reporting practices, the included studies were not suitable for meta-analysis. The quality of the evidence for patient-relevant outcomes following primary and revision hinged knee replacement was poor. We have identified several areas where study reporting could be improved in the future as described below. With respect to implant survivorship, few studies provided Kaplan–Meier survivorship estimates paired with uncertainty and numbers of patient at risk. For revision total knee replacement, there was inconsistency in the categorisation of indications for surgery, and future studies may benefit from consensus on this – for example, by using a hierarchical system for classification [37]. Only one study used a patient-completed instrument to report joint function, and future studies should look to capture this from the perspective of the patient. The Oxford Knee Score has recently been shown to be a validated instrument for the assessment of joint function after discretionary revision knee replacement [38].

It is important to identify the limitations of this study. As mentioned above, there was significant heterogeneity in the included studies, which was a contraindication to meta-analysis. The indications for rotating hinge knee replacement varied considerably, ranging from ligamentous incompetence to bony defects. The severity of disease and number of previous operations provided further sources of population diversity, and there was heterogeneity in the intervention, with a range of implants from different manufacturers utilised.

This systematic review can be used to provide some information for shared decision making with patients who are considering hinged knee arthroplasty. The revision rate following primary hinged knee arthroplasty was approximately 7% at 1 year from observational series. This is considerably higher than for primary unconstrained condylar knee arthroplasties [35]. The re-revision rate following revision hinged knee arthroplasty was higher than following primary arthroplasty. The only study that reported specifically on this outcome found a re-revision rate of 20.4% at 1 year [23]. A recent study based on data from the National Joint Registry found re-revision rates for all revision knee arthroplasties to be 19·9% at 13 years [39]. More granularity is needed on risk factors for re-revision (such as the indication for surgery) to improve communication with patients regarding the risks and benefit of hinged revision knee arthroplasty. The available evidence suggests that patients do achieve a large improvement in functional outcome following hinged knee arthroplasty for both primary and revision procedures. This systematic review was not able to identify evidence on whether surgeons should select a constrained condylar implant versus a modern rotating hinge where the patient was suitable for either device. However, we note that this is the subject of an ongoing randomized controlled trial. [40].

## Conclusion

In conclusion, the quality of the evidence for patient-relevant outcomes following hinged knee arthroplasty was poor. Prior to considering hinge TKA, patients should be counselled to expect relatively high early revision rates following both primary and revision procedures. However, when a rotating hinge TKA is indicated, our study provides evidence to support an improvement in functional outcomes after surgery.

## Data Availability

Not applicable.

## Appendix 1: Search strategy

Each database was searched from inception to January 2021. Searches were translated for each database. The search strategy for MEDLINE is provided below:

1. *Knee Prosthesis/ or Arthroplasty, Replacement, Knee/(28,369)*
2. *(total knee adj2 (arthroplast* or replacement*)).ti,ab. (22,565)*
3. *(TKA or TKR or RTKA or RTKR).ti,ab. (12,258)*
4. *1 or 2 or 3 (34,340)*
5. *(hinge* adj2 rotat*).ti,ab. (285)*
6. *RHK.ti,ab. (33)*
7. *(Kinematic adj2 (hinge* or implant* or prosthe*)).ti,ab. (69)*
8. *NexGen.ti,ab. (213)*
9. *S-ROM.ti,ab. (117)*
10. *Noiles.ti,ab. (5)*
11. *Endo-Model*.ti,ab. (37)*
12. *Finn.ti,ab. (620)*
13. *EnduRo.ti,ab. (23)*
14. *LPS.ti,ab. (82,998)*
15. *"Limb Preservation System".ti,ab. (4)*
16. *Rotaflex.ti,ab. (5)*
17. *SMILES.ti,ab. (540)*
18. *5 or 6 or 7 or 8 or 9 or 10 or 11 or 12 or 13 or 14 or 15 or 16 or 17 (84,818)*
19. *4 and 18 (461)*

## Appendix 2

See Table

**Table 4:** Summary of complication rates from each study

Author	Year	Total complications		Reoperation		Joint infection		Periprosthetic fracture		Dislocation		Patellar instability		Aseptic loosening		Prosthesis breakage		Extensor mechanism failure		Neurological injury		Superficial infection		Haematoma	
		*n*	%	*n*	%	*n*	%	*n*	%	*n*	%	*n*	%	*n*	%	*n*	%	*n*	%	*n*	%	*n*	%	*n*	%
Revision cases																									
Rodriguez	2015	1	1%	1	1%	1	1%	–	–	–	–	–	–	–	–	–	–	–	–	–	–	–	–	–	–
Neumann	2011	1	4%	1	4%	–	–	–	–	–	–	1	4%	–	–	–	–	–	–	–	–	–	–	–	–
Gudnason	2011	12	29%	12	29%	2	5%	2	5%	1	2%	1	2%	4	10%	–	–	–	–	–	–	1	2%	2	5%
Pradham	2004	–	–	–	–	–	–	–	–	–	–	–	–	–	–	–	–	–	–	–	–	–	–	–	–
Bistolfi	2013	19	36%	14	26%	3	6%	1	2%	2	4%	–	–	1	2%	6	11%	1	2%	–	–	–	–	2	4%
Barrack	2000	4	29%	1	7%			1	7%	–	–	1	7%	–	–	–	–	–	–	1	7%	–	–	–	–
Abdelaziz	2019	13	52%	13	52%	3	12%	1	4%	–	–	–	–	5	20%	–	–	–	–	–	–	–	–	–	–
Back	2008	4	14%	4	14%	3	10%	–	–	–	–	–	–	1	3%	–	–	–	–	–	–	–	–	–	–
Baier	2013	19	28%	18	26%	3	4%	1	1%	–	–	2	3%	4	6%	–	–	–	–	–	–	–	–	–	–
Bistolfi	2012	10	36%	7	25%	2	7%	1	4%	–	–	–	–	2	7%	–	–	2	7%	1	4%	–	–	1	4%
Joshi	2008	18	23%	8	10%	2	3%	–	–	3	4%	2	3%	4	5%	–	–	3	4%	–	–	–	–	–	–
Pour	2007	22	50%	19	43%	5	11%	1	2%	–	–	–	–	4	9%	–	–	–	–	–	–	–	–	8	18%
Primary cases																									
Yang	2011	7	14%	7	14%	7	14%	–	–	–	–	–	–	–	–	–	–	–	–	–	–	–	–	–	–
Rahman	2015	1	7%	1	7%	–	–	1	7%	–	–	–	–	–	–	–	–	–	–	–	–	–	–	–	–
Lozano	2012	25	23%	–	–	6	5%	2	2%	–	–	11	10%	–	–	–	–	2	2%	–	–	–	–	–	–
Kowalczewski	2014	6	50%	–	–	–	–	–	–	–	–	–	–	–	–	–	–	–	–	–	–	3	25%	3	25%
Petrou	2006	22	22%	4	4%	2	2%	3	3%	1	1%	–	–	–	–	1	1%	–	–	–	–	–	–	5	5%
Bistolfi 2	2013	29	30%	20	20%	8	8%	1	1%	5	7%	–	–	3	4%	9	13%	–	–	–	–	3	3%	–	–
Leng	2018	3	11%	2	7%	2	7%	1	4%	–	–	–	–	–	–	–	–	–	–	–	–	–	–	–	–

*n* number, % percentage of study cohort, – not reported

4.

## Appendix 3

See Table

**Table 5:** Revision rates in percent after 1 year (1 y), 5 years (5 y) and 10 years (10 y) by models reported by arthroplasty registries

	EPRD				NJR				FAR			
	*n*	1 y	5 y	10 y	*n*	1 y	5 y	10 y	*n*	1 y	5 y	10 y
NexGen Rotating Hinge (Zimmer, Warsaw, USA)	591	3.8	N/A	N/A					676	4.4	7.9	8.3
Kinematic (Stryker, Kalamazoo, USA)									119	3.4	7.9	14.0
Endo-modell (Link, Hamburg, Germany)					1278	1.4	5.3	8.7	567	3.2	7.4	12.1
EnduRo (Aesculap, Center Valley, USA)	869	4.0	N/A	N/A								
Duracon (Stryker, Kalamazoo, USA)									554	3.1	8.2	9.1
RT-Plus (Smith&Nephew, Watford, UK)	1101	4.3	N/A	N/A								
RT-Plus Modular (Smith&Nephew, Watford, UK)	327	4.8	N/A	N/A								

*EPRD* Endoprothesenregister Deutschland, *NJR* National Joint Registry, *FAR* Finnish Arthroplasty registry, *n* numbers implanted

5.
